# Shaping ability of ProTaper Universal, WaveOne and ProTaper Next in simulated L-shaped and S-shaped root canals

**DOI:** 10.1186/s12903-015-0012-z

**Published:** 2015-03-01

**Authors:** Hui Wu, Cheng Peng, Yulong Bai, Xin Hu, Lei Wang, Changyi Li

**Affiliations:** School of Stomatology, Tianjin Medical University, #12 Qi Xiang Tai Road, He Ping District Tianjin, 300070 PR China; Department of Stomatology, The Second Hospital of Tianjin Medical University, Tianjin Medical University, Tianjin, China

**Keywords:** Central axis transportation, Curvature straightening, ProTaper Universal, WaveOne, ProTaper Next

## Abstract

**Background:**

The purpose of this study was to compare the shaping ability of the ProTaper Universal (PTU; Dentsply Maillefer, Ballaigues, Switzerland), WaveOne (WO; Dentsply Maillefer) and ProTaper Next (PTN; Dentsply Maillefer) in simulated L-shaped and S-shaped root canals respectively.

**Methods:**

30 simulated L-shaped and 30 simulated S-shaped root canals in resin blocks were employed and randomly divided into 3 groups (n = 10), respectively. The canals were prepared to a tip size 25 using PTU, WO or PTN: PTU F2 (taper 0.08 over the first 3 mm from apical tip), WO Primary (taper 0.08 over the first 3 mm from apical tip), and PTN X2 (taper 0.06 over the first 3 mm from apical tip). Photos of the simulated root canals were taken pre- and postinstrumentation. The 2 layers were superimposed after a series of image processing and 10 points were selected from apical constriction with 1 mm interval. And then the central axis transportation and straightened curvature were measured with software of image analysis.

**Results:**

In simulated L-shaped root canals, PTU and PTN caused less transportation than WO at curved section (*P* < 0.05), and PTN caused the least transportation at apical constriction (*P* < 0.05). Moreover, PTN maintained the canal curvature best among the 3 groups (*P* < 0.05). But PTN produced more transportation at straight section compared with PTU and WO (*P* < 0.05). In simulated S-shaped root canals, PTN preserved the coronal curvature best (*P* < 0.05), but there was no significant difference in apical curvature since all the files straightened the curvature obviously.

**Conclusions:**

PTN showed a better shaping ability than PTU and WO at the curved section of root canals, and PTN maintained the best apical constriction. But all the files had a tendency to straighten the apical curvature in multi-curved canals.

## Background

Root canal preparation is regarded as one of the most important steps in endodontic treatment. Its main goals are to remove the infected and necrotic tissue out of root canals, to create smooth walls facilitating irrigation and obturation, to preserve the anatomy of apical foramen, and to conserve the sound root dentine for long term effect [1,2]. Nowadays, many kinds of Nickel-Titanium (Ni-Ti) rotary files have been invented to facilitate root canal preparation, such as PTU, WO and PTN. The application of these files has greatly improved cutting efficiency and safety compared with stainless steel files [3]. PTU is made of conventional Ni-Ti wire and has been widely used in root canal treatment, while both WO and PTN are made of M-wire. WO works in a reciprocating mode and finishes root canal preparation with only one file in most cases [4]. PTN is a successor to PTU. And the cross section of PTN is an off-centred rectangle which makes the file rotated in a unique asymmetric motion like a snake [5].

Shaping ability and cyclic fatigue resistance are of special importance when evaluating the performance of Ni-Ti files. Furthermore, central axis transportation and curvature straightening of root canals are two important parameters for estimating the shaping ability of Ni-Ti files. Simulated root canals in resin blocks are usually recognized as valid study models to avoid the variation among natural teeth [6], since simulated root canals could be manufactured by standardization of working length, taper, curvature, and “tissue” hardness in three dimensions [2].

To date, there have been some studies about shaping ability of PTU, WO and PTN. But the results differ from each other in separate studies. For example, Capar et al. [7] demonstrated that there was no significant difference of canal transportation and centering ratio among PTU, WO and PTN. But Yoo and Cho [8] found that WO followed the original pathway better than PTU. The possible reason for that discrepancy could be attributed to different calculation methods, even though both studies focused on analyzing the outline change of root canals to estimate the canal transportation [7,8]. Nevertheless, the present study was to acquire the central axis pre- and postinstrumentation using software of image analysis, and to directly measure the central axis transportation and curvature straightening of canals after preparation with PTU, WO and PTN. The null hypothesis was that there is no difference among the 3 rotary Ni-Ti file systems regarding the analyzed parameters.

## Methods

### Simulated root canals preparation

30 simulated L-shaped root canals (Endo Training-Bloc-L, Dentsply Maillefer) and 30 simulated S-shaped root canals (Endo Training-Bloc-S, Dentsply Maillefer) were randomly divided into 3 groups respectively (n = 10). All these canals were 0.02 taper over the 16 mm canal length. At first, #10 K-file (Dentsply Maillefer) and #13, #16 PathFile (Dentsply Maillefer) were used to glide pathway to 16 mm working length. And then, the L-shaped and S-shaped canals were prepared according to the following sequences: group PTU: #19 PathFile, PTU (SX, S1, S2, F1, F2); group WO: #19 PathFile, WO Primary; group PTN: PTN (X1, X2). And #19 PathFile was not used since PTN X1 was size 17, 0.04 taper.

During instrumentation, all simulated root canals were prepared by same experienced operator and enlarged to an apical size 25. Group PTU was prepared with a crown-down technique, while group WO and group PTN with a single-length technique recommended by the manufacturer. Each file was used in a progressive up-and-down motion within 3 times and then taken out. The canals were irrigated with distilled water until no debris was seen in the blocks. All the canals were prepared with X-Smart Plus endodontic motor and a 6:1 reduction ratio contra-angle handpiece (Dentsply Maillefer). The speed of motor was set at 300 rpm with 3 Ncm torque when PathFile, PTU and PTN were used; while the program was set at “WaveOne” mode when WO was used.

### Image processing

**Figure 1:**
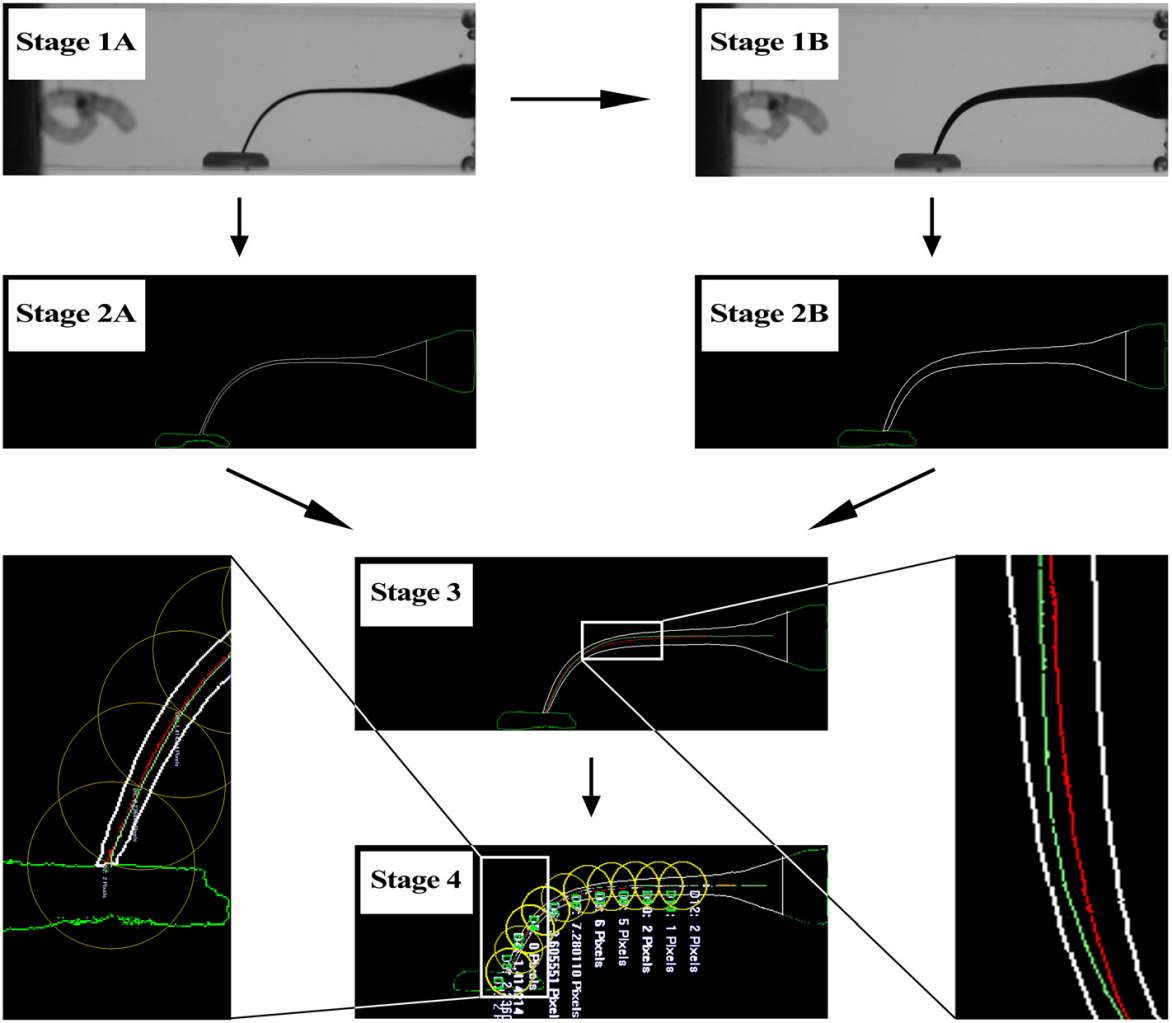
**Image processing of L-shaped canal. (Stage 1A)** the photograph was desaturated before instrumentation; **(Stage 1B)** the photograph was desaturated after instrumentation; **(Stage 2A)** the image was converted into vector one before instrumentation; **(Stage 2B)** the image was converted into vector one after instrumentation; **(Stage 3)** images pre- and postinstrumentation were superimposed into one after acquiring their central axis; **(Stage 4)** measuring the distance of central axis pre- and postinstrumentation. The green line, red line and white line represented the central axis of original root canal, the central axis of enlarged root canal, and the outline of root canal respectively.

A retainer platform for fixing a camera (Canon EOS 50D, Canon Incorporated, Tokyo, Japan) and resin blocks was made in order to take photographs pre- and postinstrumentation at the same position. Before instrumentation, black dye (Winsor & Newton, Colart Tianjin Art Materials, Tianjin, China) was filled into canals and then photographs were taken to record the shapes of original canals; after instrumentation, red dye (Winsor & Newton) was filled into canals to record the shapes of enlarged ones. When taking photographs, a file with silicon stopper was inserted into the canals as a marker. These photographs were then processed through software as follows:

1. All the photographs were inputted into software Adobe Photoshop CS6 (Adobe System, San José, CA, USA). And then they were desaturated and saved as JPEG format (Figures 1, and 2, Stage 1A and Stage 1B).

**Figure 2:**
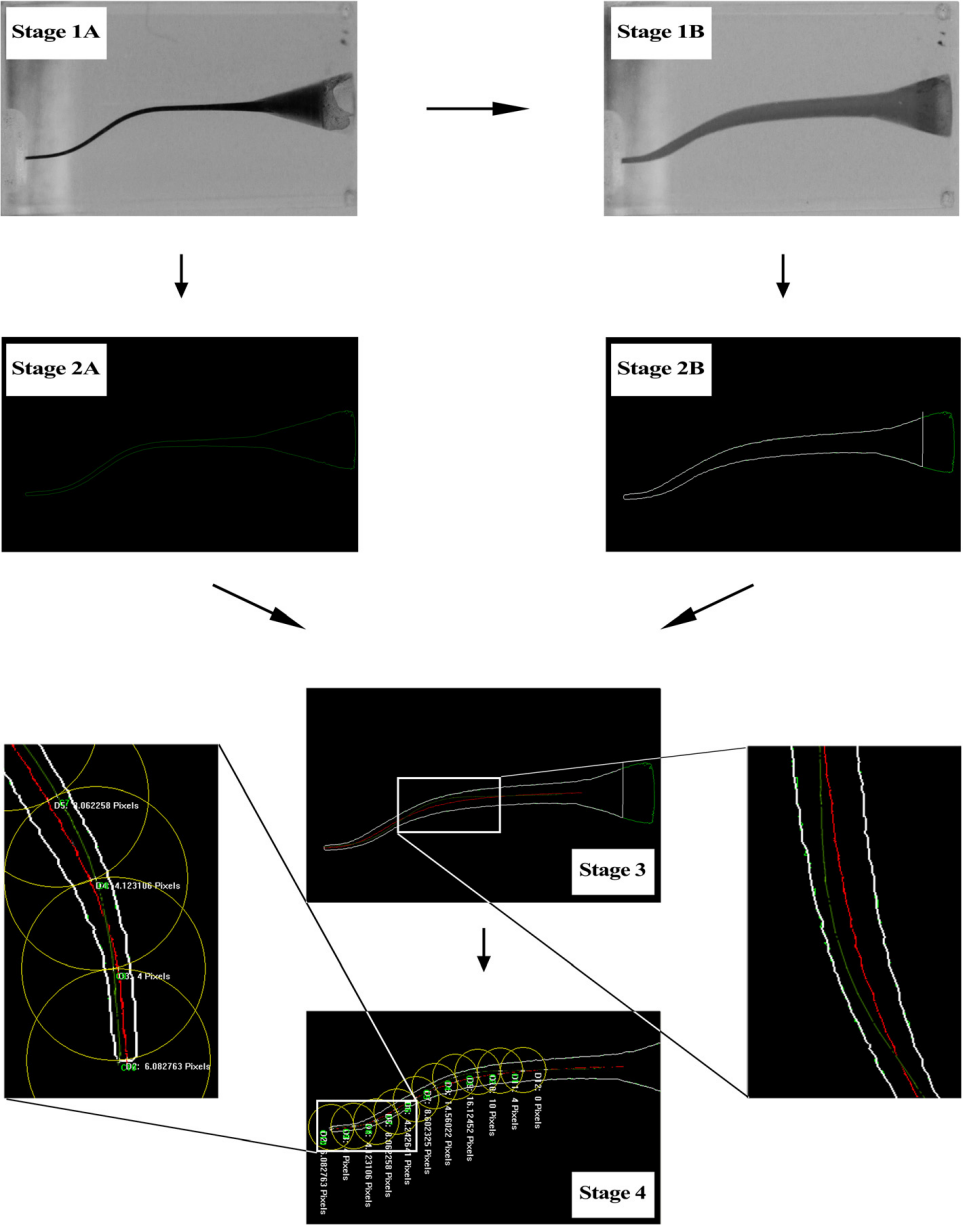
**Image processing of S-shaped canal. (Stage 1A)** the photograph was desaturated before instrumentation; **(Stage 1B)** the photograph was desaturated after instrumentation; **(Stage 2A)** the image was converted into vector one before instrumentation; **(Stage 2B)** the image was converted into vector one after instrumentation; **(Stage 3)** images pre- and postinstrumentation were superimposed into one after acquiring their central axis; **(Stage 4)** measuring the distance of central axis pre- and postinstrumentation. The green line, red line and white line represented the central axis of original root canal, the central axis of enlarged root canal, and the outline of root canal respectively.
2. The desaturated images were inputted into software Able Software R2V for Windows (Able Software, Lexington, USA) in order to converse these images into vector ones of DXF format, which facilitated accurate calculations (Figures 1, and 2, Stage 2A and Stage 2B).
3. The DXF images were inputted into software CAXA (CAXA Technology, Peking, China). With the help of CAXA, the outline of canals could easily be described. Moreover, the central axis of canals was acquired.
4. The images of original canals and enlarged ones were superimposed into one picture with the aid of software Adobe Photoshop CS6 after being dealt with CAXA. The outline of original canals was erased. Thus, the central axis of canals pre- and postinstrumentation together with the outline of enlarged canals was remained (Figures 1, and 2, Stage 3).
5. The merged images were inputted into software Image-Pro Plus 6.0 (Media Cybernetics, Warrendale, USA). Centering on apical point, the first circle was drawn with 1 mm radius. And then the next circle centered on the crossover point of the previous circle and the central axis of original canals, and so on until the 10th circle was acquired. In L-shaped canals, points 0 to 2 corresponded to the apical section, points 3 to 7 to the curved section, and points 8 to 9 to the straight section of canals. In S-shaped canals, points 0 to 4 corresponded to the apical curve, points 3 to 7 to the coronal curve [9], and points 8 to 9 to the straight section (Figures 1, and 2, Stage 4).
6. The transportation of central axis was measured based on the silicon stopper mounted on each file whose diameter was 3 mm, and defined that the left side of original central axis was negative, the right positive; the deviated angles of L-shaped canals were measured according to Schneider’ method [10]; and the S-shaped canals were measured according to Cunningham’s method [11].

### Data analysis

All these data were analyzed by IBM SPSS Statistics version 19 (SPSS China, Shanghai, China). Assuming that the populations were normally distributed and homogeneity of variance, the one-way analysis of variance could be used. Otherwise independent samples of nonparametric tests were used. The level of significance was set at *P* < 0.05.

## Results

### Central axis transportation

**Figure 3:**
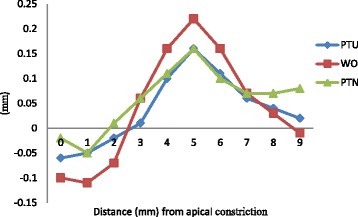
**Mean transportation of central axis after instrumentation in L-shaped root canals.** The vertical axis represented the average distance from central axis of original canals, defined that the left side of original central axis was negative and the right was positive.

In simulated L-shaped root canals, PTN caused less transportation of central axis than WO at apical section and curved section (*P* < 0.05) (Table 1 and Figure 3); and PTU also caused less transportation than WO at curved section (*P* < 0.05). Meanwhile, PTN maintained apical constriction best among the 3 groups (*P* < 0.05). But PTN produced more transportation compared with PTU and WO at straight section (*P* < 0.05).

**Table 1:** **Mean transportation ± standard deviation (in millimeters) of central axis after instrumentation at 10 points from apical constriction in L-shaped root canals**

**Group**	**0 mm**	**1 mm**	**2 mm**	**3 mm**	**4 mm**	**5 mm**	**6 mm**	**7 mm**	**8 mm**	**9 mm**
PTU	0.06 ± 0.03^b^	0.06 ± 0.03^a^	0.06 ± 0.04^a,b^	0.05 ± 0.04^a^	0.10 ± 0.06^a^	0.16 ± 0.05^a^	0.11 ± 0.04^a^	0.06 ± 0.04^a^	0.04 ± 0.04^a^	0.05 ± 0.04^a^
WO	0.10 ± 0.03^b^	0.11 ± 0.03^b^	0.07 ± 0.04^b^	0.06 ± 0.04^a^	0.16 ± 0.04^b^	0.22 ± 0.04^b^	0.16 ± 0.03^b^	0.07 ± 0.02^a^	0.03 ± 0.01^a^	0.02 ± 0.01^a^
PTN	0.05 ± 0.02^a^	0.07 ± 0.04^a^	0.06 ± 0.02^a^	0.06 ± 0.04^a^	0.11 ± 0.03^a^	0.16 ± 0.02^a^	0.10 ± 0.02^a^	0.07 ± 0.02^a^	0.07 ± 0.03^b^	0.08 ± 0.03^b^
P value	<0.05	<0.05	<0.05	>0.05	<0.05	<0.05	< 0.05	>0.05	< 0.05	< 0.05

Within the same column, values with same superscript letter were not statistically different.

**Figure 4:**
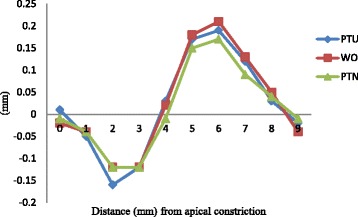
**Mean transportation of central axis after instrumentation in S-shaped root canals.** The vertical axis represented the average distance from central axis of original canals, defined that the left side of original central axis was negative and the right was positive.

In simulated S-shaped root canals, all the files straightened the curvatures significantly (Table 2 and Figure 4). Moreover, group PTU deviated from the central axis further than the other groups at 2 mm (*P* < 0.05).

**Table 2:** **Mean transportation ± standard deviation (in millimeters) of central axis after instrumentation at 10 points from apical constriction in S-shaped root canals**

**Group**	**0 mm**	**1 mm**	**2 mm**	**3 mm**	**4 mm**	**5 mm**	**6 mm**	**7 mm**	**8 mm**	**9 mm**
PTU	0.08 ± 0.04^a^	0.07 ± 0.05^a^	0.16 ± 0.04^b^	0.12 ± 0.05^a^	0.05 ± 0.06^a^	0.17 ± 0.05^a^	0.19 ± 0.04^a^	0.12 ± 0.05^a^	0.05 ± 0.04^a^	0.06 ± 0.03^a^
WO	0.06 ± 0.04^a^	0.06 ± 0.03^a^	0.12 ± 0.03^a^	0.12 ± 0.05^a^	0.05 ± 0.04^a^	0.18 ± 0.06^a^	0.21 ± 0.06^a^	0.13 ± 0.05^a^	0.06 ± 0.03^a^	0.04 ± 0.04^a^
PTN	0.08 ± 0.07^a^	0.06 ± 0.04^a^	0.12 ± 0.05^a^	0.12 ± 0.04^a^	0.04 ± 0.03^a^	0.15 ± 0.06^a^	0.17 ± 0.07^a^	0.09 ± 0.06^a^	0.06 ± 0.04^a^	0.04 ± 0.03^a^
P value	>0.05	> 0.05	<0.05	>0.05	>0.05	>0.05	>0.05	>0.05	>0.05	>0.05

Within the same column, values with same superscript letter were not statistically different.

### Curvature straightening

In simulated L-shaped root canals, the original angle was 30 degree. And PTN maintained the canal curvature best (*P* < 0.05) while PTU straightened the curvature most (*P* < 0.05) (Table 3).

**Table 3:** **Mean values ± standard deviation of straightened degree from original angles after instrumentation in L-shaped and S-shaped root canals**

	**In L-shaped canals**	**In S-shaped canals**	
**Group**	**Straightened angles (°)**	**Coronal curvature (°)**	**Apical curvature (°)**
PTU	6.00 ± 1.09^c^	6.32 ± 0.80^b^	22.51 ± 3.45^a^
WO	4.73 ± 0.54^b^	7.31 ± 0.73^c^	20.44 ± 2.02^a^
PTN	0.90 ± 1.58^a^	5.19 ± 1.42^a^	21.03 ± 2.21^a^
*P* value	<0.05	<0.05	>0.05

Within the same column, values with same superscript letter were not statistically different.

In simulated S-shaped root canals, the original angle of coronal curvature was 20 degree and the apical one was 30 degree. PTN maintained the coronal curvature best (*P* < 0.05) while WO straightened the coronal curvature most (*P* < 0.05). But all files straightened the apical curvature visibly and there was no significant difference among them (*P* > 0.05) (Table 3).

## Discussion

The present study compared the shaping ability of PTU, WO and PTN in simulated L-shaped and S-shaped root canals. The simulated L-shaped root canals of 30 degree were severely curved canals [10] and the S-shaped were multi-curved [12]. The null hypothesis was rejected. The results of the present study showed that in severely curved canals, PTN caused less transportation at apical section and better maintained canal curvature, even though PTN produced more transportation at straight section compared with PTU and WO; in multi-curved canals, PTN caused the least coronal curvature straightened, but all the files straightened the apical curvature. In both types of canals, the great transportation appeared at the corresponding curved sections, and all the files tended to pull curved canals into straight ones.

Multiple factors can affect the shaping ability of Ni-Ti files such as alloy microstructure, taper, cross-sectional geometry, movements and system composition. So far, there are mainly 3 phases of microstructure of Ni-Ti wire: austenite, martensite, and R-phase. Ni-Ti alloy displays strong and hard when in austenite phase and it displays flexible and ductile when in martensite phase [13]. The microstructure of PTU is mostly consisted of austenite [14], while WO and PTN are newly invented files whose microstructure is mainly consisted of martensite [15]. And PTU straightened the canal curvature most in severely curved canals.

American Dental Association defined the taper of endodontic files as 0.02 in 1981, and allowed the variation within 0.05 mm in 2001 [16]. So there are 3 types of tapers: constant taper, progressive taper (from apical to coronal) and decreasing taper [17,18]. It is claimed that progressive taper increases the flexibility of files while decreasing taper makes files much stiffer [19]. For PTU, S1 and S2 have a progressive taper, while F1 and F2 have a decreasing taper [17]. SX is designed to flare root canal orifice, S1 to prepare the coronal one-third of root canals, S2 to prepare the middle one-third, F1 and F2 to prepare the apical one-third and further enlarge the middle one-third of root canals. For WO, WO primary has a decreasing taper. For PTN, X1 and X2 have a progressive taper at the apical section while a decreasing taper at the coronal section [20]. The progressive taper of PTN makes it more flexible than PTU and WO at the apical section. Thus, PTN caused the least transportation at apical section in severely curved canals.

Each file system has benefits and weaknesses. Cross-sectional geometry of Ni-Ti files are various such as triangle, rectangle, slender-rectangle, or square. Some studies find that files with square cross section have the highest screw-in force and flexural stiffness followed by the rectangular ones, the triangular ones and the slender-rectangle ones [21,22]. PTU has a cross section of convex triangle [23]. WO changes cross sections over the working length from a modified convex triangle in the tip region to a convex triangle similar to PTU near the shaft [24]. And PTN has an off-centred rectangular cross section which makes the files rotated in a unique asymmetric motion like a snake [5]. Therefore, PTN, the rectangular cross section together with a decreasing taper at the coronal section, had higher screw-in force and flexural rigidity than PTU and WO, which resulted in more transportation at the straight section in severely curved canals.

Up to now, there have been two sorts of file system composition, that is, single-file system and multi-file system. Single-file system usually associates with reciprocating motions (ie, WO and Reciproc), while multi-file system with continuous rotation (ie, PTU and PTN). It is demonstrated reciprocation has better performance than continuous movements [25]. But the present study exhibited that WO produced more transportation at curved parts than PTU and PTN in severely curved canals. That is probably because the single-file system with sharp cutting edges could provide high cutting efficiency, which brings about more canal transportation than multi-file system.

## Conclusions

According to the study, PTN could cause less transportation at apical section and better maintain the canal curvature than PTU and WO in severely curved canals. In addition, PTN could better preserve the coronal curvature than PTU and WO in multi-curved canals, although all the files straightened the apical curvature visibly.

## Abbreviations

PTU: ProTaper Universal
WO: WaveOne
PTN: ProTaper Next
Ni-Ti: Nickel-Titanium
